# Ventilator integrated polygraphy for patients using non-invasive ventilation; Case report

**DOI:** 10.3389/fmed.2022.852896

**Published:** 2022-07-25

**Authors:** Helena López-Brull, Estefanía Mira-Padilla, Sarah Hussein, Antoine Guerder, Estelle Wozniak, Violeta Esteban-Ronda, Jésus Gonzalez-Bermejo

**Affiliations:** ^1^Hospital General Universitario Alicante, Alicante, Spain; ^2^Hospital Universitario Reina Sofía, Córdoba, Spain; ^3^Service de Réhabilitation Respiratoire (Département R3S), AP-HP, Groupe Hospitalier Universitaire APHP-Sorbonne Université, Site Pitié-Salpêtrière, Paris, France; ^4^Hospital Universitario San Juan de Alicante, Alicante, Spain; ^5^Institut National de la Santé et de la Recherche Médicale (INSERM), UMRS1158 Neurophysiologie Respiratoire Expérimentale et Clinique, Sorbonne Université, Paris, France

**Keywords:** NIV, COPD, patient ventilator asynchrony, monitoring, case report

## Abstract

The COVID-19 pandemic has meant that home respiratory services have needed to be reviewed. As a result, new solutions have been developed and implemented. The Vivo 45™ (Breas, Mölnlycke, Sweden) is a ventilator that offers clinicians the ability to attach effort belts to the device. This allows the clinician to review ventilator traces with the addition of thoracic and abdominal activity. This allows more flexibility for the monitoring of patients at home and in the hospital, with detection of patient ventilator asynchrony (PVA). Decreasing PVA may improve ventilator adherence and increased ventilator usage improves survival. We report three cases of patients undergoing overnight monitoring with the Vivo 45™, highlighting the benefit of ventilator integrated polygraphy. In our three cases we demonstrate a simple safe tool to optimize NIV treatment over one or two-night recordings using ventilator downloaded software with the addition of effort belts and pulse oximetry without involving more than one machine and without hospitalization in a sleep unit.

## Introduction

Non-invasive ventilation (NIV) is a very effective treatment for patients with chronic diseases such as chronic obstructive pulmonary disease (COPD), overlap syndrome, obesity hypoventilation or neuromuscular diseases. NIV has been shown to correct arterial blood gases and improve survival (1–3). However, in order to do this it is needed to use the ventilator for at least 4 h per day (4–8). The tolerance of the treatment depends on a correct adaptation of the patient to the ventilator and the minimization of unintentional leaks. Optimizing ventilation to reduce patient ventilator asynchrony improves survival (9) and improves adherence (10). Therefore, it is very important to monitor patients for patient ventilator asynchrony (PVA) and identify the type of asynchrony in order to optimize the treatment. Polysomnography is a theoretical gold standard but is not routinely available in many centers so polygraphy is often performed to evaluate PVA. However, treatment can also be evaluated with regards to patient tolerance and effectivity by analyzing the ventilatory traces captured by inbuilt programs in the machine (11). These traces can be downloaded from the ventilator to a software program on a personal computer (PC) or viewed in some devices via the cloud. This feature allows the clinician to review the ventilator traces at any time. Nevertheless, these programs don't give us as much information as a polygraphy can. In addition to the ventilator waveforms, polygraphy has the additional benefit of looking at abdominal and thoracic chest wall movement. This in turn provides us with additional accurate information about unintentional leaks, partial or total upper airway obstruction with or without a reduction of ventilatory drive (12). Recent technological drives, partially as a result of the COVID-19 pandemic, and trends to more home monitoring have led to advances in technology. This has resulted in the Vivo 45™ ventilator (Breas, Mölnlycke, Sweden). This device allows connection of two accessory belts (one abdominal and one thoracic), along with oximetry and capnography sensors. This data can then be reviewed in PC software (Breas Medical, Mölnlycke, Sweden), performing something very close to a polygraphy. As yet no one has reported the clinical benefit of the addition of abdominal and thoracic belts in conjunction to conventional ventilator memory download in improving PVA. We report three cases, in whom consent was obtained to share their information anonymously for education purposes, in which ventilator integrated polygraphy improved patient care by identifying PVA in review of ventilator download traces, and this permitted us to change the ventilator settings in order to correct the PVA.

## Case report

**Figure 1:**
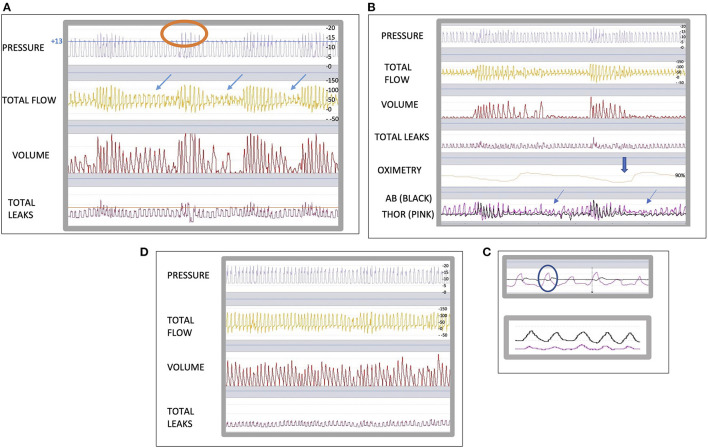
**(A)** shows a 5 min ventilator download traces in Breas PC software (Breas Medical, Mölnlycke, Sweden). The leaks were in its majority in the intentional range for the type of interface. The arrows indicate the decrease in flow. To note an overshoot of the pressure during the patient's triggered cycles (circle). **(B)** shows an 5 min epoc downloaded to Breas PC software (Breas Medical, Mölnlycke, Sweden) with the addition of SpO2 monitoring and thoracic [THOR (pink)] and abdominal [AB (black)] belts connected to the ventilator. The bold thick arrow shows an important desaturation and the thin arrow shows an obstructive event with persistent ventilatory drive. **(C)** shows the thoracoabdominal trace in 1 min epoc: in the upper image we can see opposition of phases, marked with a circle, with the abdominal band (black) slightly weaker, and we can compare it to the lower image were we can see normal thoracoabdominal movements. **(D)** shows a 5 min epoc in Breas™ PC software (Breas Medical, Mölnlycke, Sweden). Note, we no longer observe obstructive events.

Patient A, is a 75 year-old male with a diagnosis of severe COPD, increased body mass index (BMI) 38.5 kg/m2. Despite his high BMI he was not known to have obstructive sleep apnoea (OSA). Our patient presented with a diaphragmatic hernia and had undergone recent abdominal surgery. During the post-operative recovery he experienced severe hypoventilation. 15 days post operation he requires long-term NIV and was referred to our unit. On the day of entry to our unit, his arterial blood gases (ABG's) showed a PaCO2 of 61 mmHg (normal range 35–45 mmHg). He was initiated with a domiciliary NIV device, in pressure-support ventilation, spontaneous timed (ST) mode. His settings were; inspiratory positive airway pressure (IPAP) 23 cmH2O, expiratory positive airway pressure (EPAP) 6 cmH2O, back up respiratory rate (RR) 12 breaths per minute (bpm) and inspiratory time (Ti) minimum 0.5 and maximum 1.5 s. Unfortunately, he experienced high unintentional leaks and this made it impossible to correct his nocturnal ventilation and improve his PaO2 and PaCO2. Therefore, we reduced the IPAP to 13 cmH2O and the EPAP to 5 cmH2O in order to help decrease the unintentional leak that was not resolved with various mask changes. We downloaded the internal ventilator memory data and saw a decrease in unintentional leaks during the night. An example of the ventilator download can be seen in Figure 1A. There is no unintentional leak but, we observed a decrease in flow and the pressure remained unvaried. From this information (ventilator flow traces alone), we are unable to know whether we are looking at an upper airway closure, or a reduction of the respiratory drive with glottic closure (12), and if there is any oxygen desaturation as a result of the events. Therefore, to obtain more information, we connected an oximeter and abdominal and thoracic bands to his Vivo 45 ventilator. The ventilator flow traces with the additional monitoring were downloaded and showed a persistent ventilatory drive, as we can identify the thoracoabdominal movements; phase opposition of the abdominal and thoracic belts (Figure 1C, upper image); slight decrease in abdominal activity at the same time as the decrease in flow (Figure 1B); decrease in oxygen saturation. These findings reflect that Patient A, is trying to overcome upper airway obstruction. To note, we can see that the patient is mainly utilizing his accessory muscles because of his diaphragm weakness (inverse opposition phase in the bels, with abdominal belt decreasing as thoracic belt increases). The slight decrease in abdominal activity can be explained by the recent abdominal surgery. We therefore increased the EPAP by 1 cmH2O, keeping the same Pressure Support (PS) and subsequent evaluation of the traces revealed a decrease in obstructive events (Figure 1D).

**Figure 2:**
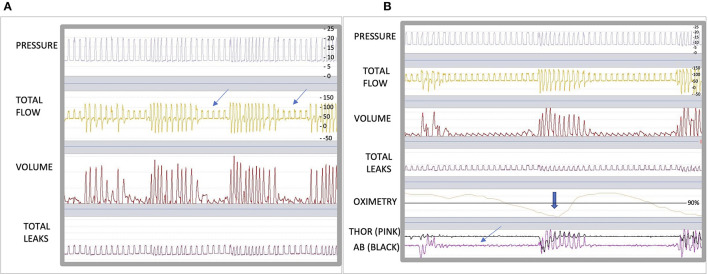
**(A)** shows a 5 min epoc in Breas™ PC software (Breas Medical, Mölnlycke, Sweden). The arrows highlights obstructive events. **(B)** shows a 5 min epoc in Breas™ PC software with the addition of SpO2 probe and abdominal [AB (black)] and thoracic [THOR (pink)] belts. The bold arrow shows important desaturation and the thin arrow shows the absence of movement of belts.

Patient B, is a 68 year-old woman who underwent neck surgery, complicated with a diaphragmatic dysfunction resulting in nocturnal hypoventilation. This manifested as severe orthopnea requiring NIV. At our center she was initiated with a Vivo 45 ventilator in pressure-support ventilation in ST mode. Her settings were: IPAP 19 cmH2O, EPAP 8 cmH2O, Ti minimum 0.8 and maximum 1.8 s and back up RR 14 breaths per minute. After the initiation of NIV, her ABG showed a PaCO2 of 45 mmHg. Downloaded overnight ventilator traces showed an absence of leaks and a decrease in flow (Figure 2A). We questioned the origin of this and its possible consequences (upper airway obstruction with persistent ventilatory drive or glottic closure with a reduction in central ventilatory drive?) (12, 13). To help answer these questions with more information, we connected an oximeter and abdominal and thoracic bands to her ventilator. We downloaded the ventilator traces along with the additional monitoring. In these traces, we noticed an absence of movement of the thoracoabdominal belts, followed by desaturation (Figure 2B). This is because there is a decrease in the respiratory drive, likely to be due to ventilatory-induced hyperventilation. We therefore adjusted the setting by decreasing the pressure support, which normalized the NIV accordingly.

**Figure 3:**
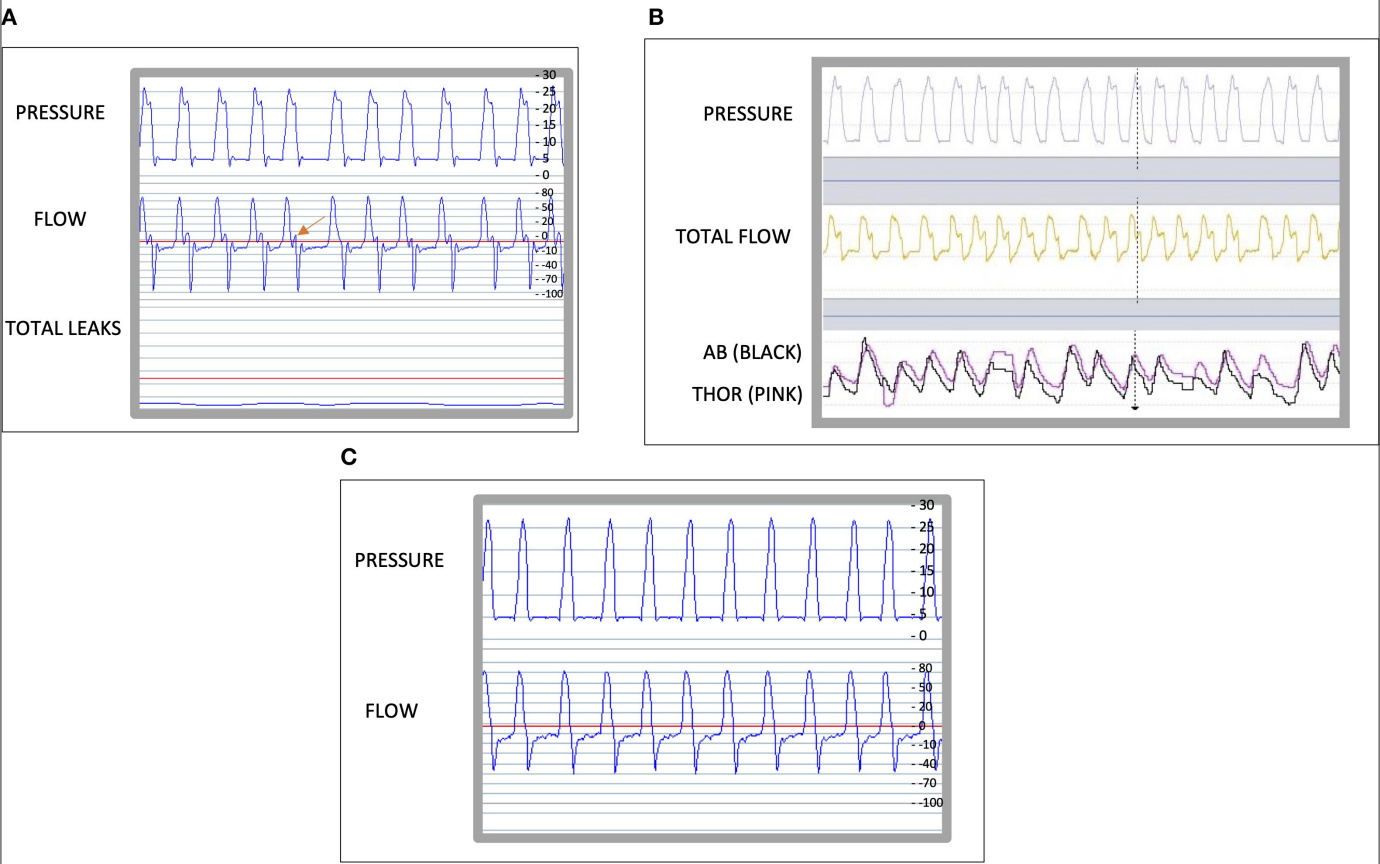
**(A)**, Shows a 1 min epoc of the traces of a Lumis 150 ventilator (Resmed, San Diego, CA, USA) using Rescan program (ResMed, Lyon, France). The leak is close to 0l/mn. The arrow indicates the asynchrony. **(B)** Shows a 1 min epoc of the traces on a Breas ™ PC software showing the abdominal [AB (black)] belt and thoracic belt [THOR (pink)], the dotted vertical line shows a late cycling. **(C)** shows a 1 min epoc of the traces of a Resmed ™ ventilator using Rescan software.

**Figure 4:**
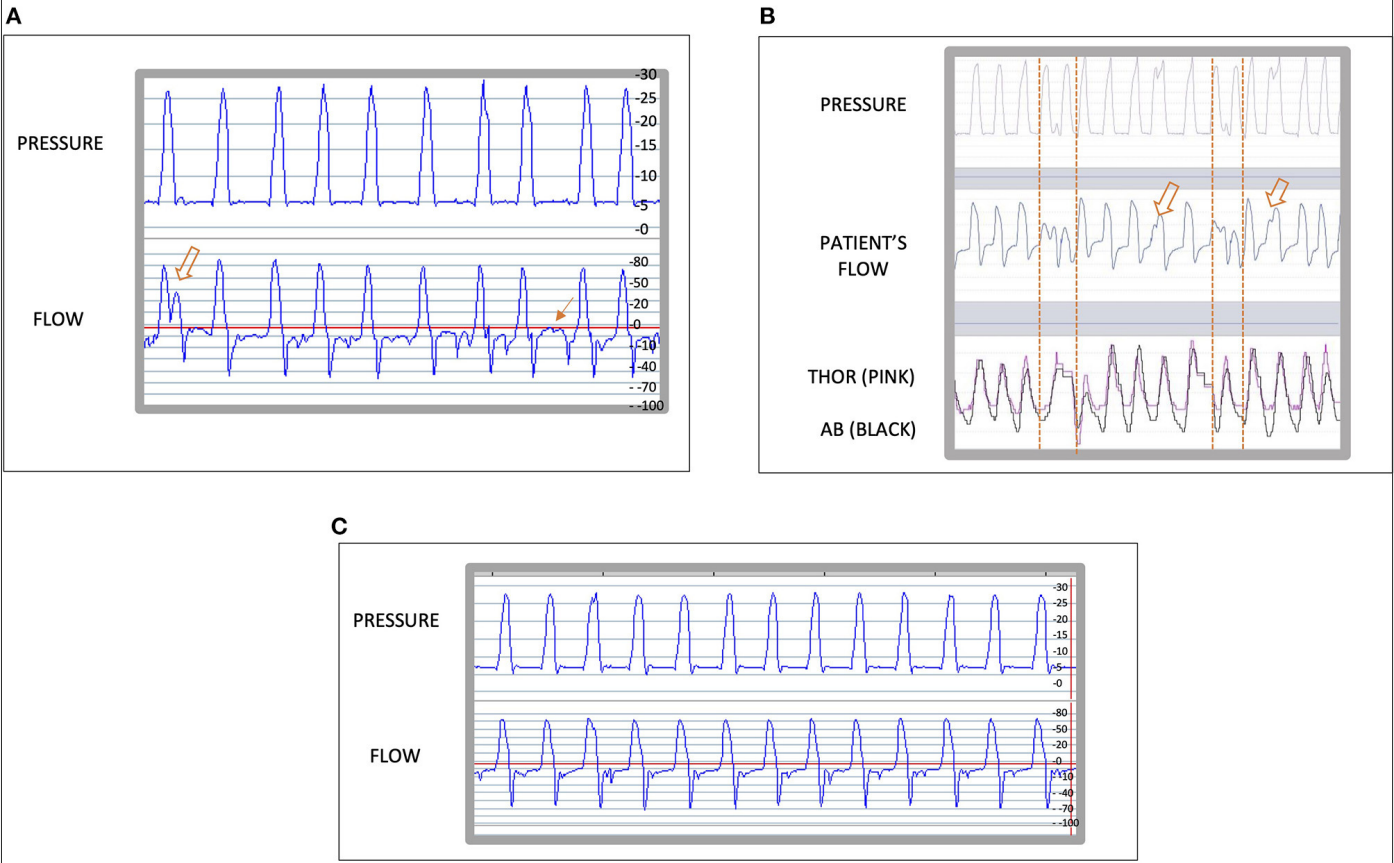
**(A)** shows a 1 min reading of the traces of a Lumis 150 ventilator (Resmed, San Diego, CA, USA) using Rescan program (Resmed, San Diego, CA, USA). The arrows indicate patient ventilator asynchrony. **(B)** shows a 1 min epoc in Breas™ PC software (Breas Medical, Mölnlycke, Sweden). Two intracycle effort with deformation of the pressure curve are shown with a hollow arrow and two doble triggering are shown between two dotted vertical lines. These, are signs of “air hunger.” **(C)** shows a 1 min epoc of the traces of the Lumis 150 ventilator using the Rescan program.

Patient C, is a 51 year-old male with a known diagnosis of bronchiectasis, thoracic distention and chronic hypercapnic respiratory failure, requiring long-term NIV. He was initiated with a Lumis 150 ventilator (Resmed, San Diego, CA, USA) in pressure-support ventilation, ST mode. His settings were, IPAP of 22 cm H2O and EPAP 8 cm H2O, inspiration trigger medium, cycling trigger medium and Timin 0.8 s and Timax 1.5 s. An ABG was performed after the initiation of NIV showing a PaCO2 of 81 mmHg. When the ventilator traces were downloaded and reviewed using the Rescan™ (Resmed, San Diego, CA, USA), an intracycle asynchrony was noted (14) (Figure 3A), This made us want to establish its nature. We hypostasized that it could either be a late cycling, an intracycle effort made by the patient or as a result of a non-compliant lung. To help us identify the cause, we transferred the patient onto a Vivo 45 with the same treatment settings and connected an oximeter and abdominal and thoracic belts. The following morning we downloaded the ventilator traces with the additional monitoring. We clearly identified a late cycling of the device because, the thoracoabdominal movements change to expiration before the ventilator finishes delivering the breath in (Figure 3B). The settings were changed on the patient's own Lumis 150 ventilator by, decreasing the Ti minimum. The following morning we downloaded the ventilator traces, we found the late cycling had been resolved (Figure 3C). However, we noted, different PVAs (Figure 4A) that we could interpret as either late cyclings or intracycle efforts. We wanted to be sure if these could be signs of underassitance, as we could also see an ineffective effort. Once again, we used the Vivo 45 with the additional monitoring and identified some intracycle efforts, shown by an inspiration movement starting in the middle of a cycle and, as a new finding, there also appeared double triggering, which is shown as two cycles in the pressure and flow traces triggered by only one respiratory effort. All these PVA suggest “air hunger” (Figure 4B). Thus, we increased his IPAP by 2 cmH2O which led to the asynchronism to disappear (Figure 4C). The patient had been acclimatized to the Lumis 150 device and did not want to change device preferring to keep the Lumis 150.

## Discussion

We believe these cases identify the importance of the addition of polygraphy to the ventilator for a correct adaptation of the settings. In our three patients, polygraphy combined with a non-invasive ventilator download was shown to be very useful in the diagnosis of new events and to evaluate the efficacy of the treatment in our three patients. The addition of integrated pulse oximetry and thoracic and abdominal belts allowed us to identify both the cause of the obstructions in two of our cases as well as their severity, as they lead to a desaturation, which we could have not seen in a simple trace. In the first case we identified a significant obstructive event with persistence of the respiratory drive, that we resolved by increasing the EPAP. In the second case we were able to identify a glottic closure caused by hyperventilation, which if it wasn't for the abdominal and thoracic belts, we would not have been able to differentiate it from an obstructive event and we would probably have exacerbated it by increasing the inspiratory pressure. In the last case we were able to identify 3 different asynchronies, that we wouldn't have been able to see without the abdominal and thoracic belts, by making the necessary adjustments we were able to increase the patient's adherence.

Some experts (12, 14) and some societies as the Swiss Society of Pulmonology (15) recommend that polysomnography or polygraphy should be performed when there is a suspicion of asynchrony or in the presence of nocturnal hypoventilation despite optimization. They also recommend a low threshold to carry out polysomnography or polygraphy when using automated modes of NIV because most of these modes have not been independently validated or proven to be superior to manual titration (15). However, this often requires admission to a sleep unit for this to be performed. Our patients attend a chronic ward specializing in NIV without a direct attachment to the sleep unit. Therefore, we are very familiar with looking at ventilator traces, this experience means that ventilator integrated polygraphy could easily be performed on the ward. Centers with less expertise will need to be trained on identification of events in order to utilize this tool in the best possible way. However, we believe the adaptation of NIV settings to for the individual patient is crucial and necessary for recovery. Like Hannan and co-workers (10) we believe it will increase ventilator usage at home, and potentially increase survival. Daily monitoring of these patients was carried out by reading the ventilatory traces given by the inbuilt ventilator programs, however, sometimes, additional information was needed. Indeed, Georges and co-workers (16), evaluated different methods of monitoring (ABG, nocturnal SpO2, TcPCO2, and data provided by built-in software) to improve NIV efficacy. They concluded that monitoring ABG and nocturnal SpO2 is not enough to assess NIV efficacy and combining data from ventilator built-in software and transcutaneous carbon dioxide was the best strategy to detect poor NIV efficacy. However, the addition of abdominal and thoracic effort belts has the potential to further enhance treatment efficacy by accurate detection of apnea events and PVA (12, 14). Table 1 describes the different events that can be detected by adding the effort belts to our ventilator. It is important to note that not all device internal software detect events in the same way (17). The addition of abdominal and thoracic effort belts allows accurate detection of events (12, 14). The current system is an open-loop system. The clinician needs to adjust de ventilator settings after identification of PVA. Clinicians are often very busy and are not be able to monitor the PVA in real time. Progression of this work should be to develop a closed-loop system to adjust the ventilator appropriately aiming to irradicate PVA to help clinician workload and improve patient care.

**Table 1:** Types of assynchrony.

**Asynchrony/event**	**Description in a polygraphy**
Double triggering	Where there are two cycles are noted on the pressure and flow curves but, only one cycle on the abdominal belt
Auto-trigering	At a given time, a cycle appears on the pressure and flow curves but, there is no thoracoabdominal movement
Ineffective efforts	There are movements recorded on the thoracoabdominal belts which indicate inspiratory efforts from the patient. But, you do not see any ventilator response as there is no change in the flow and pressure curves
Uncoupling	Respiratory rate of the ventilator and that of the patient are clearly different
Delayed cycling	The patient's expiration showed by the belts, starts when the ventilator remains still in inspiration (showed by the flow and pressure curves)
Premature cycling	The demand of the patient, showed by the thoracoabdominal belts, continues after the ventilator has already cycled into expiration (showed by the flow and pressure curves)
Upper airway obstruction	Is depicted by a decrease in the flow curve, with phase opposition between thoracic and abdominal belts. This is because there is an in increase in effort to overcome the obstruction
Decrease of ventilatory drive	Decrease in the flow curve as well as in the amplitude of the traces of the thoracoabdominal belts

*Table shows, on the left column, a list of all the types of events and PVA that can be identified by ventilator integrated polygraphy. On the right column it shows a brief description of each event*.

The addition of effort belts to a ventilator allowing for ventilator integrated polygraphy within the same device is a novel concept and negates the need for separate equipment. This can enable clinicians to carry out this monitoring in the ward or the home. Patients can either have this device long-term and attach extra monitoring for one or two nights to ensure optimal adaption of the ventilator or the device can just be used for titration. This may therefore be a useful tool in centers who do not have easy access to attended manual titration studies. Further work in this area is therefore warranted (18).

In conclusion, these three cases demonstrate that it is possible to optimize a NIV treatment over one or two-night recordings using a very simple and safe tool, without involving more than one machine and without hospitalization in a sleep unit. Complete monitoring at home can be even available now thanks to these tools.

## Data availability statement

The original contributions presented in the study are included in the article/supplementary material, further inquiries can be directed to the corresponding author/s.

## Author contributions

HL-B, EM-P, and EW contributed in the download, reading and interpretation of the ventilator, and polygraphy flow traces. SH, AG, and JG-B contributed in the treatment and follow up of the patients in the hospitalization ward. HL-B wrote the first draft of the manuscript. JG-B, VE-R, and EM-P contributed in the manuscript editing. All authors contributed to manuscript revision, read, and approved the submitted version.

## Conflict of interest

The authors declare that the research was conducted in the absence of any commercial or financial relationships that could be construed as a potential conflict of interest.

## Publisher's note

All claims expressed in this article are solely those of the authors and do not necessarily represent those of their affiliated organizations, or those of the publisher, the editors and the reviewers. Any product that may be evaluated in this article, or claim that may be made by its manufacturer, is not guaranteed or endorsed by the publisher.
